# P07-07 Put the promotion of physical activity for people with non-communicable diseases on the political agenda in Luxembourg!

**DOI:** 10.1093/eurpub/ckac095.107

**Published:** 2022-08-29

**Authors:** Alexis Lion, Anne Frisch, Lucienne Thommes, Arno Bache, Anik Sax, Romain Seil, Axel Urhausen, Daniel Theisen, Charles Delagardelle

**Affiliations:** 1 Fédération Luxembourgeoise des Associations de Sport de Santé, Strassen, Luxembourg; 2 Luxembourg Institute of Research in Orthopedics, Sports Medicine and Science, Luxembourg, Luxembourg, Luxembourg; 3 Centre Hospitalier de Luxembourg, Luxembourg, Luxembourg; 4 Medizinische Sport Gruppen für Personen mit Orthopädischen & Metabolischen Störungen, Luxembourg, Luxembourg; 5 Fondation Cancer, Luxembourg, Luxembourg; 6 Ministère de la Santé, Luxembourg, Luxembourg; 7 Oeuvre Nationale de Secours Grande-Duchesse Charlotte, Leudelange, Luxembourg; 8 ALAN Maladies Rares Luxembourg, Bascharage, Luxembourg

**Keywords:** physical activity, promotion, healthcare, politics, non-communicable diseases

## Abstract

**Issue/problem:**

In 1984, cardiologists and cardiac patients created an association offering physical activity (PA) for people with cardiovascular diseases in Luxembourg (0.6 million inhabitants). During the last 20 years, several associations created therapeutic PA for people with a wide range of non-communicable diseases (NCDs). Today more than 70 hours of therapeutic PA are weekly offered. Nevertheless, the organization of these PA is incomplete and not enough patients benefit from it.

**Description of the problem:**

Sustainability of privately organized courses is challenging. Despite a governmental financial support, the organization of PA offer remains mainly based on the idealism of a limited number of volunteers. However, this kind of commitment is disappearing and jeopardizes a correct offer of therapeutic PA. Only a minority of physicians are referring their patients on a regular basis and only a minority of them are engaging in an active lifestyle.

**Results:**

A project was launched in 2013 to compile, monitor and promote the therapeutic PA offered by different associations. As a result of this project, six associations created a sport federation in 2016 destined to improve the organization of therapeutic PA for people with NCDs. In 2018, the federation obtained an increase in the financial support from the Ministry of Health. The same year, a campaign promoted the therapeutic PA but had no impact on the number of patients counselled about therapeutic PA (26.6%) and on the physician's knowledge of the therapeutical PA offer (21%). The federation is now trying to develop and implement deeper actions, such as a PA referral scheme.

**Lessons:**

The collaborative and synergetic work of the different associations offering PA for people with NCDs bundled their activities resulting in an increased consideration and support from the Ministry of Health. Nevertheless, structural improvements should be conducted to increase sustainably the number of physically active patients.

**Main messages:**

- The promotion and the organization of a valuable and sustainable therapeutic PA for patients with NCDs must be professionalized and publicly supported.
- A larger strategic vision needs to be developed by the government and healthcare providers to organize and support preventive medicine in Luxembourg.

